# Low Dose Gamma Irradiation of *Trypanosoma evansi* Parasites Identifies Molecular Changes That Occur to Repair Radiation Damage and Gene Transcripts That May Be Involved in Establishing Disease in Mice Post-Irradiation

**DOI:** 10.3389/fimmu.2022.852091

**Published:** 2022-05-13

**Authors:** Richard T. Kangethe, Eva M. Winger, Tirumala Bharani K. Settypalli, Sneha Datta, Viskam Wijewardana, Charles E. Lamien, Hermann Unger, Theresa H.T. Coetzer, Giovanni Cattoli, Adama Diallo

**Affiliations:** ^1^ Animal Production and Health Laboratory, FAO/IAEA Agriculture and Biotechnology Laboratory, IAEA Laboratories Seibersdorf, International Atomic Energy Agency (IAEA), Vienna, Austria; ^2^ Biochemistry, School of Life Sciences, University of KwaZulu-Natal, Pietermaritzburg, South Africa; ^3^ UMR CIRAD INRA, Animal, Santé, Territoires, Risques et Ecosystèmes (ASTRE), Montpellier, France

**Keywords:** *Trypanosoma evansi*, gamma irradiation, TryMS array, vaccine, surra

## Abstract

The protozoan parasite *Trypanosoma evansi* is responsible for causing surra in a variety of mammalian hosts and is spread by many vectors over a wide geographical area making it an ideal target for irradiation as a tool to study the initial events that occur during infection. Parasites irradiated at the representative doses 100Gy, 140Gy, and 200Gy were used to inoculate BALB/c mice revealing that parasites irradiated at 200Gy were unable to establish disease in all mice. Cytokine analysis of mice inoculated with 200Gy of irradiated parasites showed significantly lower levels of interleukins when compared to mice inoculated with non-irradiated and 100Gy irradiated parasites. Irradiation also differentially affected the abundance of gene transcripts in a dose-dependent trend measured at 6- and 20-hours post-irradiation with 234, 325, and 484 gene transcripts affected 6 hours post-irradiation for 100Gy-, 140Gy- and 200Gy-irradiated parasites, respectively. At 20 hours post-irradiation, 422, 381, and 457 gene transcripts were affected by irradiation at 100Gy, 140Gy, and 200Gy, respectively. A gene ontology (GO) term analysis was carried out for the three representative doses at 6 hours and 20 hours post-irradiation revealing different processes occurring at 20 hours when compared to 6 hours for 100Gy irradiation. The top ten most significant processes had a negative Z score. These processes fall in significance at 140Gy and even further at 200Gy, revealing that they were least likely to occur at 200Gy, and thus may have been responsible for infection in mice by 100Gy and 140Gy irradiated parasites. When looking at 100Gy irradiated parasites 20 hours post-irradiation processes with a positive Z score, we identified genes that were involved in multiple processes and compared their fold change values at 6 hours and 20 hours. We present these genes as possibly necessary for repair from irradiation damage at 6 hours and suggestive of being involved in the establishment of disease in mice at 20 hours post-irradiation. A potential strategy using this information to develop a whole parasite vaccine is also postulated.

## Introduction

*Trypanosoma evansi*, a mechanically transmitted haemoparasitic flagellate from the genus *Trypanosoma*, is geographically the most widely dispersed member of the group and is found in Asia, South America, the Middle East, and Africa, with occasional outbreaks in parts of Europe (1). *T. evansi* has a wide host range and infects many animal species causing Surra in cattle, buffalo, pigs, and donkeys amongst other domesticated animals and is a significant cause of morbidity and mortality in camel populations (1–5). Zoonotic cases have also been reported in humans and are referred to as atypical human trypanosomosis (a-HT) (6). Some a-HT infections were found in individuals that have a fully functional apolipoprotein L1 (ApoL1), the serum lytic factor that prevents African Animal Trypanosomosis (AAT) in humans, suggesting that the parasite is capable of employing other mechanisms that are yet to be elucidated for survival in the human host (7–9). *Trypanosoma evansi* parasites are transmitted by blood sucking flies, which in addition to the tsetse fly, include the horsefly, stable fly, horn fly, and deerfly (*Tabanus* and *Stomoxys* spp.) and by vampire bats in South America (10–12). Infection occurs when an infected fly, a temporary host with parasite contaminated mouthparts, is able to feed on several uninfected mammalian hosts thus quickly establishing disease. The combination of having multiple host animals and a wide range of mechanical vectors has enabled the parasite to spread out of tsetse-infected areas onto four different continents (13, 14).

The symptoms associated with *T. evansi* infections differ depending on the susceptibility of the infected host and include anemia, fever, loss of weight and productivity, as well as abortion (15–17). Like all other extracellular parasites in the genus, *T. evansi* primarily evades mammalian host immunity by switching its variable surface glycoprotein (VSG). At the onset of the infection, initial VSG-induced Th1 responses are followed by T cell exhaustion, altered antigen presentation, defective complement activation and eventually the destruction of the bone marrow, marginal zone, and follicular B cell populations, thus eliminating B cell memory and making the host vulnerable to other secondary diseases (9, 18–21). Vaccinated pigs and water buffalos perform poorly against already vaccinated diseases after acquiring *T. evansi* infections (9, 21). All of these combined factors have hindered the development of potential vaccines against all trypanosome species to date.

There is experimental evidence to show that some animals can undergo self-cure from *T. evansi* infection, an event that is accompanied by qualitative and quantitative changes to their lymphocyte populations (22–24). Studies in buffalo in Indonesia have also shown that in some naturally infected animals, there is a long-term development of immunity that could be enhanced by the use of suitable vaccination methods (25). Approaches to trypanosome vaccine research have included using recombinant subunit targets as antigens. Structural recombinant antigens studied include tubulin, the GPI anchor of VSG, and more recently, an invariant flagellum antigen protein that was successful in mice but with no further developments for affected livestock (26–30). Other promising antigens developed include different Trypanosome proteases as ‘anti-disease’ targets that alleviate symptoms rather than neutralizing the parasite, but difficulties were encountered in the field due to their complex chemistries (31–36).

Initial studies using irradiation as a tool for developing trypanosome vaccines were made in the early 1970s by destroying the parasite with high irradiation doses of up to 1000Gy (37). Animals inoculated with irradiated parasites developed good humoral immune responses against VSG and were protected against homologous trypanosome challenge but not heterologous variants (37–45). The irradiation doses used were however lethal to the parasite and much higher than those used by current related irradiated parasite vaccines. Previous attempts to develop trypanosome vaccines with irradiation were made using 600Gy, a dose that is four times higher than what had been successful in the development of an irradiated plasmodium vaccine at 150Gy (46). For the non-dividing but metabolically active Schistosoma irradiated vaccine, the effective dose used was between 200- and 500Gy (47). Other studies that measure the effect of using irradiation on the trypanosome spp. have been carried out on *T. cruzi* due to its high tolerance to high doses of irradiation of up to 1500Gy (48–50). Successful developments in malaria have also demonstrated the feasibility of utilizing irradiation as a technique for developing vaccines (46, 51–53). Other potential irradiated parasite vaccines include those for schistosomiasis (47, 54), parasitic bronchitis caused by *dictyocaulus* spp. (55, 56) and Babesia (55, 56).

Following the success of malaria and other parasite irradiated vaccines, we hypothesized that irradiated, metabolically active, but non-replicating parasites could be used as a vaccine. To this end, *T. evansi* was chosen as the representative parasite due to its wide geographical host and vector range with little developmental differences between the vector and mammalian parasite forms. The possibility of restricted strain diversity and the transient nature of infection with *T. evansi* in different transmission vectors, implies that the mechanisms for infection used by the parasite would be universal for all trypanosome spp. Parasites were irradiated at different doses ranging from 100 - 250Gy so that they were temporarily unable to multiply but still capable of synthesizing many of the proteins that could potentially help the parasite establish disease in mice. Irradiated *T. evansi* parasites were subsequently observed *in vitro* before inoculation and challenge of mice. Doses that produced living and non-infectious parasites were then studied using a whole transcript gene microarray and compared to that of infectious parasites. Data was analyzed to identify genes and processes that are responsible for radiation damage repair and may possibly be involved in enabling the parasites to establish an infection in the mammalian host after recovery.

## Methods

### Trypanosome Culture

Bloodstream forms of *T. evansi* RoTat 1.2 wild-type parasites obtained from the Institute of Tropical Medicine, Antwerp, Belgium were isolated in 1982 from a buffalo in Indonesia (ITMAS #020298) . Parasites were cultured in supplemented Iscove’s Modified Dulbecco’s Medium (IMDM) as previously described (57). Briefly, 1 liter of IMDM containing 3.6 mM NaHCO_3_ (Thermo Fischer Scientific, Roskilde, Denmark) was supplemented with 1 mM hypoxanthine, 1 mM sodium pyruvate, 0.16 mM thymidine, 0.05 mM bathocuprone sulfate, 1.0 mM L-cysteine and 0.2 mM 2-mercaptoethanol). The pH was adjusted between 7.2 and 7.4 and 10% (v/v) heat inactivated fetal calf serum (Gibco, Paisley, UK) was added before filtration using a 0.2 µm filter. Parasites were seeded at 2 x 10^5^ and regularly split at mid logarithmic growth phase. Bloodstream forms of *T. evansi* Can 86K were also obtained from the Institute of Tropical Medicine and were isolated in 1986 from a dog in Brazil (ITMAS #140799B) and used for heterologous challenge studies (58).

### Trypanosome Irradiation Parameters

Bloodstream forms of *T. evansi* RoTat 1.2 wild-type parasites (1x10^6^ mid log phase) were resuspended in complete IMDM in a 50 mL conical tube filled to the top with no air bubbles and put on ice. Parafilm was used to avoid spillage and contamination. Parasites on ice were then placed in the Gamma irradiator and exposed to the Cobalt 60 source for the calculated length of time it took to deliver the desired dose (Model 812 Co-60 irradiator, Foss Therapy Services, Inc., California, USA). Initial doses ranging from 100Gy to 600Gy were carried out and later on restricted to 100Gy - 250Gy with 20Gy intervals. Irradiated parasites were then pelleted and finally resuspended at 2x10^5^ parasites per ml with 1 ml dispensed per well in a 24 well flat-bottomed culture plate and *in vitro* growth was observed. Initial doses of 100Gy, 120Gy, 140Gy, 160Gy, 180Gy, 200Gy, and 250Gy were applied to identify the D10 dose value which is the dose required to reduce parasite load by 90% or 1 log, (59).

### Cell Proliferation Assay

Bloodstream forms of *T. evansi* RoTat 1.2 wild-type parasites (1x10^7^ mid log phase) were pelleted and re-suspended in 1 mL IMDM containing 5% (v/v) FCS. A volume of 1.1 µL of CFSE stock solution (5 mM 5(6)-Carboxyfluorescein diacetate N-succinimidyl ester; Sigma Aldrich, St. Louis, MO, USA) was mixed with 110 µL of PBS and added to the parasites before gently mixing by inverting the tube. Parasites were incubated with CFSE at 37°C for 5 minutes with gentle mixing 3 to 4 times. The CFSE-labelled parasites were then resuspended in 10 mL IMDM and washed three times with IMDM to remove excess CFSE dye before transfer to a 50 mL conical tube filled to the top with IMDM and used for irradiation at the desired dose on ice. Irradiated CFSE-labelled parasites were then pelleted and finally resuspended at 2x10^5^/mL with 1 mL dispensed per well in a 24 well flat-bottomed plate. Cell proliferation was measured using flow cytometry immediately after staining and every 24 hours until the culture was overgrown or had stopped dividing. Flow cytometry data were acquired using the Gallios flow cytometer (Beckman Coulter, USA) and analyzed with Kaluza software (Beckman Coulter, USA). Cell populations were gated by forward and side-light scatter parameters, as shown in **Supplementary Figure 1**.

### Mouse Infections

In order to observe what role irradiation plays in *T. evansi* virulence and infection, four groups of 8-week-old BALB/c female mice (8 per irradiation dose) were inoculated twice by intraperitoneal injection using an insulin syringe with 1 ×10^4^ *T. evansi* RoTat 1.2 irradiated parasites per mouse resuspended in 50 µl of phosphate buffered saline (PBS). A control group was also infected with 1 × 10^4^ *T. evansi* RoTat 1.2 wild type non-irradiated parasites along with a second challenge control group that received 50 µl PBS alone. The two inoculations were carried out at day 0 and day 14. On day 28 post inoculation, surviving mice were challenged with heterologous 1 × 10^3^ *T. evansi* Can 86K. A parallel experiment where mice were challenged with homologous 1 × 10^3^ *T. evansi* RoTat 1.2 wild type non-irradiated parasites was also performed. Parasitemia was measured on alternative days by bleeding from the tail and the survival and wellbeing of the mice was monitored. Parasite load was estimated in each inoculated mouse using the rapid matching method as previously described (60). Blood samples measured for parasitemia were blinded to the readers. Plasma samples were also collected from the different groups of mice using heparinized capillary tubes over the course of infection and stored at -80°C for further cytokine analysis. Mice were sourced and housed at the University of Veterinary Medicine in Vienna. Infection and care of the infected mice was carried out using protocols approved by the institutional ethics committee of the University of Veterinary Medicine, Vienna, and the national authority according to § 26 of the Austrian Law for Animal Experiments, Tierversuchsgesetz 2012-TVG 2012 under the No. GZ 68.205/0069-WF/II/3b/2014.

### Mouse Bio-Plex Cytokine Assay

A custom 10-plex cytokine assay (Bio-Plex: Bio-Rad, Hercules, USA) was used to quantify the plasma levels of 10 cytokines in mouse (Mo) plasma [interferon-gamma (IFN-γ), tumor necrosis factor-alpha (TNF-α), interleukin-1 alpha (IL-1α), IL-1β, IL-4, IL-6, IL-10, IL-12 (p40), IL-12 (p70) and IL-13] as described by the manufacturer. Briefly, a 1 in 4 dilution of plasma collected from the different groups of mice during inoculation (days 0 to 28) was incubated with beads coupled to monoclonal antibodies specific for each component of the cytokine panel. Samples were washed before adding detection antibodies and developed for reading using the Bio-Plex^®^ 200 suspension array system. Absolute interleukin concentrations were calculated using Bio-Plex Manager™ software and samples were analysed using a one-way ANOVA with Dunn’s Multiple Comparison Test in GraphPad Prism 5 for each cytokine. Boxplots were generated using ggplot2 package in R (61).

### *Trypanosome* spp. Whole Transcript Gene Array Design (TrypMS)

A custom *Trypanosome* spp. array that covers the genomes of three trypanosome species, *T. brucei*, *T. evansi* and *T. congolense* was designed by Affymetrix (Santa Clara, California, USA) with input from the authors. Briefly, an expression/tilling array request form for TrypMS (Trypanosoma multi species) was completed describing features desired according to the Affymetrix MyGeneChip™ design guide. The request was sent to the design team along with four Trypanosome genomes (*T. brucei Lister 427, T. brucei TREU 927, T. evansi STIB 805* and *T. congolense IL3000*). All genomes were downloaded from the TriTrypDB datadase (https://tritrypdb.org/tritrypdb/app; accessed on 29.10.2014). A proposal was then prepared by Affymetrix and confirmed. The final array designed contained the fully sequenced *T. brucei* genome as a backbone with selected *T. evansi* and *T. congolense* sequences that do not overlap with the *T. brucei* genome. Approximately 94.9% of *T. evansi* genome is identical to the reference *T. brucei* genome (62). A total of on average 9,300 whole gene transcripts from all three species were targeted with most having 8 to 25 probe pairs per gene. The array was produced using the 16 sample arrays per plate format. Microarray data produced from this experiment is available in the ArrayExpress database (http://www.ebi.ac.uk/arrayexpress) under accession number E-MTAB-11705.

### Measurements of RNA Abundance Using the Affymetrix^®^ Whole Transcript Array TrypMS

In order to characterize irradiated parasites, RNA extracted from three or more replicates each at 1, 6, and 20 hours after irradiation at the different indicated doses (0, 100, 140, 200, and 250Gy) was used for hybridisation onto the TrypMS array. RNA extraction was carried out using a RNeasy kit (Qiagen, Hilden, Germany). Extracted RNA was then processed through several amplification cycles, including first-strand cDNA synthesis using a poly dT primer, second-strand cDNA synthesis, *in-vitro* cRNA (copy RNA) synthesis, and final second-cycle single strand cDNA synthesis using GeneChip™ WT PLUS reagent kit by Affymetrix. Single strand cDNA generated was fragmented, labelled, and hybridized to the *Trypanosome* spp. whole transcript array before processing using the Gene Titan^®^ Multi-Channel (MC) instrument from Affymetrix. Intensity CEL files generated after hybridisation and scanning were analysed using Affymetrix^®^ Expression console software and files that passed all quality control parameters were converted into CHIP files that were subsequently interpreted using Affymetrix^®^ Transcriptome Analysis Console (TAC3) Software. Lists of genes were prepared using *T. brucei* annotation with figures on fold change (FC), ANOVA *p-value* and false discovery rate (FDR) adjusted *p-value* assigned to each gene described.

### Gene Ontology Analysis

Gene transcript abundance affected by irradiation at different doses after 6 hours and 20 hours were used to query TriTrypDB for gene ontology (GO) enrichment data (63). GO enrichment terms identified were used together with fold changes calculated from the TrypMS array to calculate a Z score using tools from GOplot on R (64). The Z score is a value that indicates whether the GO term associated with a set of genes [biological process (BP)/molecular function (MF)/cellular components (CC)] is more likely to be decreased or increased and is calculated as *Z = (up* ***-*** *down)/SQRT (Total)* where *up* and *down* are the number of assigned transcripts up-regulated (logFC > 0) or down- regulated (logFC < 0) in the data set, divided by the square root of the total number of genes associated with the identified GO term. Calculated Z scores versus -log adj *p* values were used to make combined bubble plots (with BP, MF and CC) for 100Gy, 140Gy, and 200Gy at 6 hours and 20 hours post-irradiation. A GO circle plot for the top 10 GO enrichment terms with the most significant *p* values was also plotted for 100Gy at 6 hours and 20 hours. GO chord plots that display the relationship between genes and GO terms was plotted for the ten most significant terms for 100Gy at 20 hours. Gene ontology analysis and visualization was performed using customized python codes and the GO plot manual (http://wencke.github.io/).

### Confirmatory Quantitative PCR

Total RNA previously harvested from parasites 1, 6, or 20 hours after irradiation was used to generate copy DNA (cDNA) using random hexamer primers and a Superscript II reverse transcriptase kit (Invitrogen). The synthesized cDNA was used as a template for confirming fold-changes for 17 downregulated and 5 upregulated gene transcripts previously identified using the TrypMS array by relative quantification using TERT as an internal control as previously described (65). The primer sequences used for amplification are listed in **Supplementary Table 1**.

## Results

### *In Vitro* and *In Vivo T. evansi* RoTat 1.2 Irradiated Parasite Dynamics

**Figure 1:**
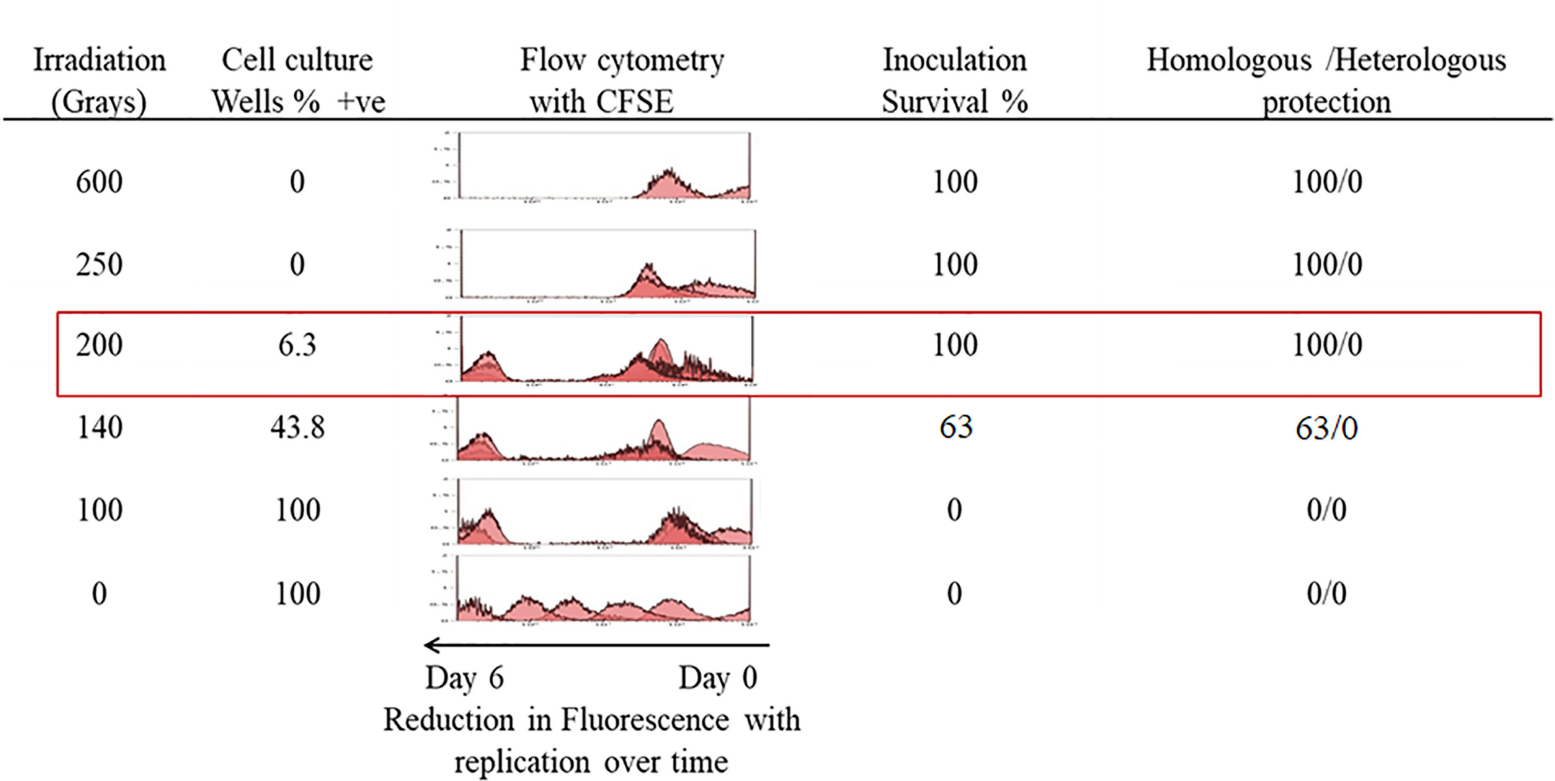
*In-vitro* growth analysis of irradiated parasites. Parasites irradiated at different doses were observed over a 7-day period post-irradiation and 200Gy was identified as the maximum dose applicable. Percentage survival was calculated from the number of wells containing parasites that survived in a 24 well plate. A parallel plate was used to measure replication of parasites labelled with CFSE, which halves in concentration with each doubling of parasites. Eight female BALB/c mice per group were each inoculated with 1 x 10^4^ parasites irradiated with doses ranging from 600Gy to 100Gy and a control group received 1 x 10^4^ non-irradiated parasites. Surviving mice were further challenged with 1 x 10^3^ of either homologous *T. evansi* RoTat 1.2 or heterologous Can 86K strains.

Our initial experiments were carried out to determine the doses at which *T. evansi* parasites survive irradiation but are rendered non-infectious in a mouse infection model. Parasites irradiated at doses above 200Gy did not survive post-irradiation and no cultures were viable after approximately 3 days when irradiated at 250Gy and on average 24 h at 600Gy (**Figure 1**). Less than 10% of parasites (1-2 wells per 24 well plate) irradiated at 200Gy survived 7 days post-irradiation. However, once the parasites survived the week post-irradiation, they were able to divide rapidly and reach log-phase growth rates. Close to a half (43.8%) of the wells plated with parasites irradiated at 140Gy also survived post-treatment. Parasites irradiated at 100Gy all survived treatment and apart from a short period of no division for approximately 48 h, and all wells with parasites, once again, became viable cultures. The D10 dose value for the irradiation of the parasites was calculated as 19.83Gy (**Supplementary Figure 2**). The 100Gy, 140Gy, and 200Gy irradiated parasites were selected for further analysis to represent a deeper range of doses tested (**Supplementary Figure 3**). A CFSE assay that measures replication of cells, confirmed our visual observations by mirroring a similar trend in parasite numbers. Parasites receiving doses above 200Gy were able to divide two to three rounds before death. For the doses at 200Gy and below, the recovery time for irradiated parasites was extended the closer the dose was to 200Gy before the parasites were capable of replication (**Figure 1**).

**Figure 2:**
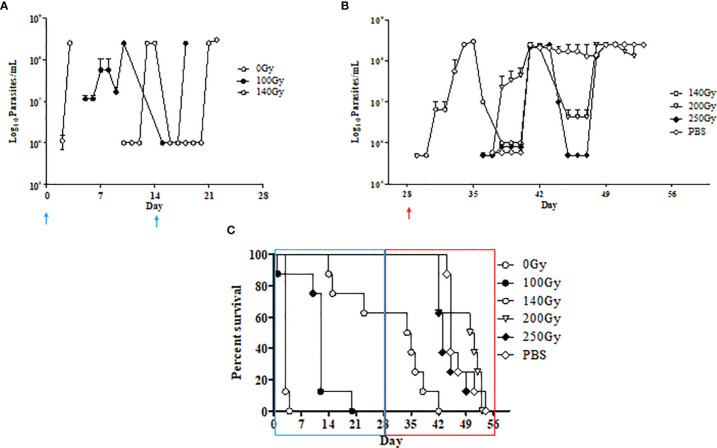
*In-vivo* characteristics of irradiated parasites at different doses. **(A)** Parasite counts in mice that developed an infection after inoculation with *T. evansi* RoTaT1.2 irradiated parasites on days 0 and 14 (↑) are plotted. Mice in groups inoculated with either 0Gy or 100Gy irradiated parasites all developed parasitemia although with a delayed prepatent period at day 4 for mice inoculated with 100Gy irradiated parasites. Only three mice in the group inoculated with 140Gy irradiated parasites were infected with parasites first appearing at day 9. **(B)** Parasite counts in mice that developed an infection after heterologous challenge with *T. evansi* Can 86K on day 28 (↑). **(C)** Kaplan Meier survival analysis shows a 15-day gap between mice inoculated with 0Gy and 100Gy irradiated parasites and the remaining 5 mice in the group inoculated with 140Gy irradiated parasites succumbing faster to heterologous challenge on day 28 compared to the remaining groups.

**Figure 3:**
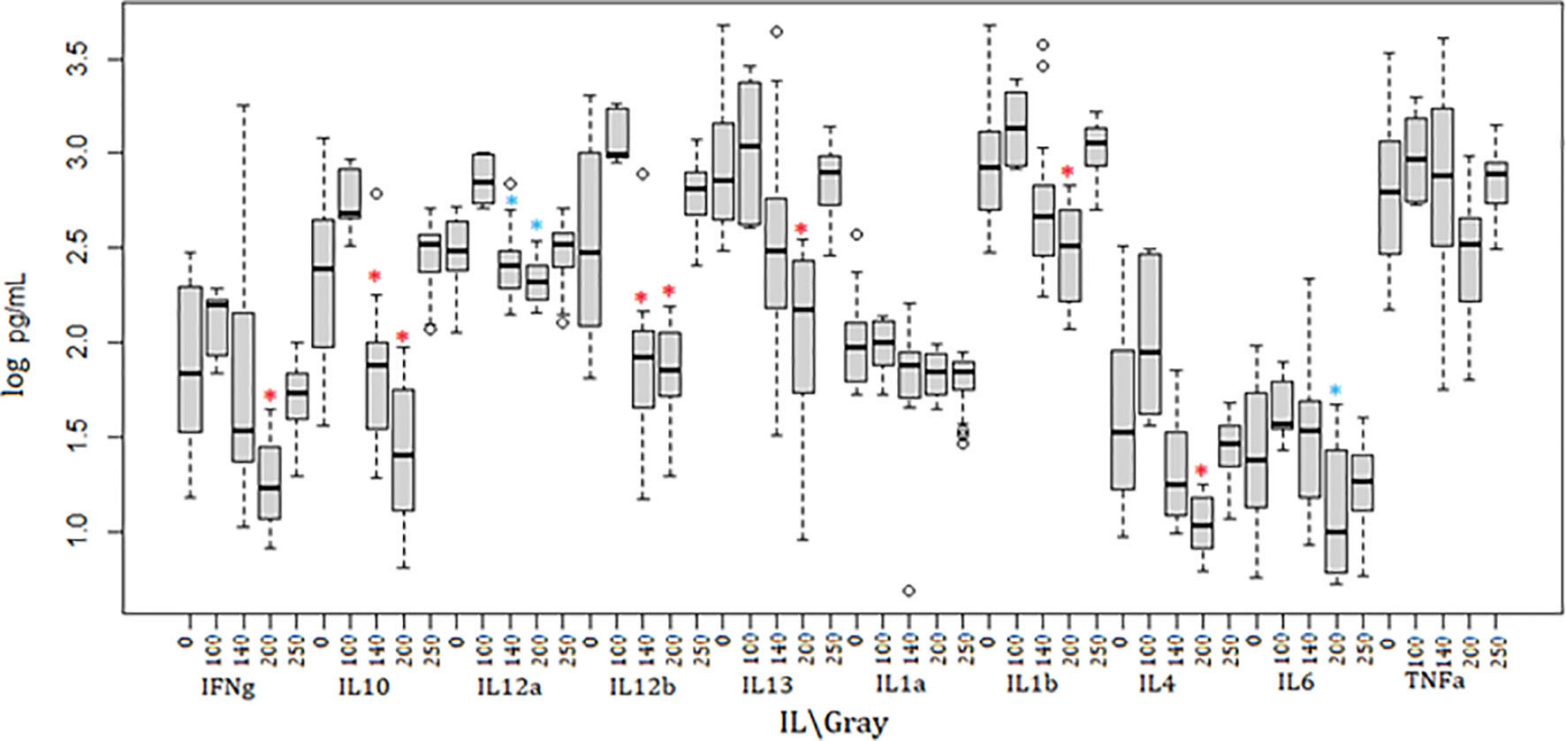
Cytokine dynamics in mice immunized using irradiated parasites. Ten different cytokines were measured through the course of inoculation and challenge. Mice inoculated with 200Gy irradiated parasites had significantly lower levels of IFNγ, IL10, IL12b, IL13, IL1b and IL4 when compared to 0Gy (*). IL12a and IL6 were significantly lower when comparing 200Gy to 100Gy irradiated parasite inoculation (*). Significantly lower IL10, IL12a and IL12b were also observed in mice inoculated with 140Gy irradiated parasites (*). Analyses were calculated using one-way ANOVA for each cytokine, *p < 0.01.

Irradiated parasites were also used for *in vivo* studies (**Figure 2**). Mice from the group inoculated with parasites irradiated at 100Gy all succumbed to death after developing parasitemia levels similar to the control group that received 0Gy parasites (**Figures 1**, **2A, C**). However, the 100Gy group had a longer prepatent period than the control group, with parasites first appearing on day four rather than day two (**Figure 2A**). The three mice that developed parasitemia in the group inoculated using 140Gy irradiated parasites had an even longer prepatent period with infection first observed after day eight when compared to both 0Gy and 100Gy groups at two and four days, respectively. One of the mice in the 140Gy group showed parasitemia only after the second inoculation and thereafter quickly succumbed to infection (after day 21, **Figure 2A**). All other inoculated mice did not develop any detectable parasitemia even after homologous challenge (results not shown). Heterologous challenge, however, resulted in parasitemia in all groups. The remaining five mice in the 140Gy group developed parasitemia and succumbed much faster than the remaining 200Gy and 250Gy inoculated groups (**Figures 2B, C**). The two groups of mice inoculated with 200Gy and 250Gy irradiated parasites did not show any significant difference compared to the PBS inoculated challenge control group. Plasma samples collected from mice during inoculation revealed that the 200Gy inoculated group displayed statistically significant depressed levels of IFNγ, IL10, IL12b, IL13, IL1b, IL4, when compared to control mice (0Gy, **Figure 3**). The group of mice inoculated using 100Gy irradiated parasites showed similar cytokine levels as the control mice, apart from where levels were significantly higher when compared to groups inoculated with 140Gy and 200Gy irradiated parasites for IL12a and IL6 in the group inoculated with 200Gy irradiated parasites (**Figure 3**).

### Gene Transcript Abundance Is Dependent on and Differentially Affected by the Irradiation Dose Applied

**Figure 4:**
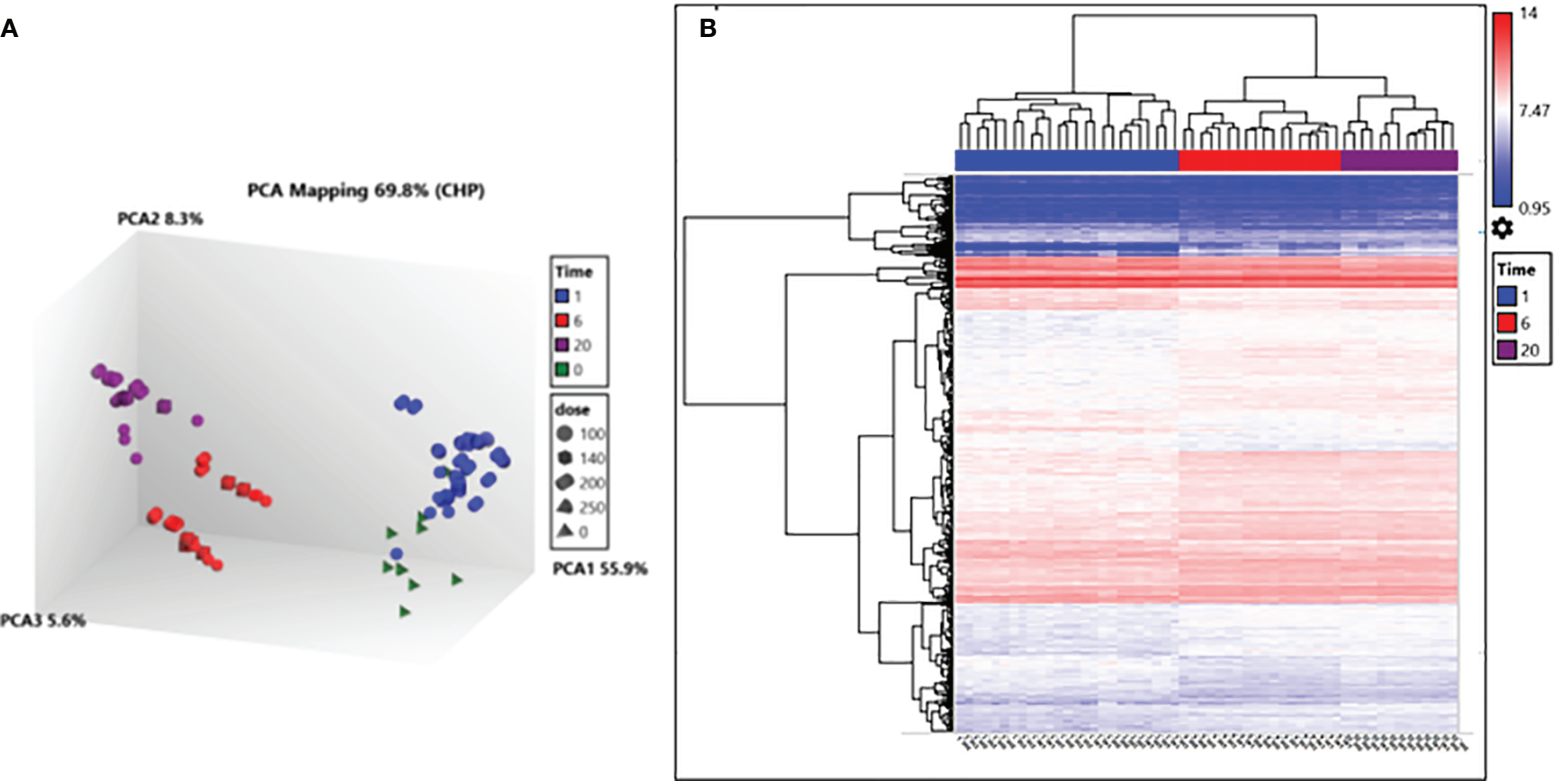
Transcription analysis of irradiated parasites using the TrypMS array. **(A)** PCA mapping separated samples according to time post-irradiation. **(B)** Hierarchical clustering of deferential gene transcripts according to time and further sub clustered using the gene ID. Fold change (FC) cut-off points of > 2 and < 2 were used as the limits for upregulation and downregulation respectively and a gene-level P-value < 0.05.

Parasites analysed for differential gene transcript abundance after irradiation treatment could only be reliably deciphered after RNA was extracted 6 hours and 20 hours post-irradiation. This was clear after principal components analysis (PCA) of RNA isolated from irradiated parasites processed using TrypMS (**Figure 4A**). All 20 hour and 6 hour delay extracted samples clustered individually and separately from 1 hour delay and non-irradiated 0Gy extracted samples clustered close to each other. Hierarchical clustering of differentially transcribed RNA from 1 hour, 6 hour and 20 hour delay samples separated accordingly as displayed (**Figure 4B**). Samples from the non-irradiated group (0Gy) were used as the baseline for hierarchical clustering.

**Figure 5:**
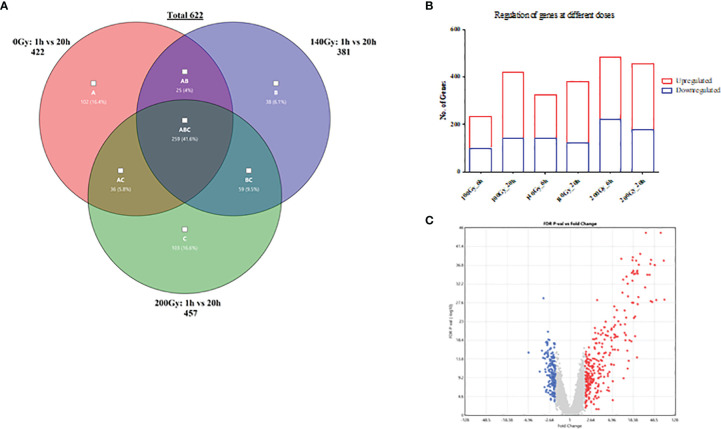
General differences in gene transcript changes according to dose and time post-irradiation. **(A)** Bar graph showing the ratio of gene transcript abundance upregulated to downregulated at different doses at 6 and 20 hours post-irradiation. **(B)** Volcano plot of fold-change (FC) vs false discovery rate (FDR) *p* value for 200Gy irradiated parasites at 20h. **(C)** Venn diagram describing the distribution of 622 differential gene transcripts at 20 hours after irradiation with 259 genes common across all three doses when compared to 1 hour after irradiation, 102, 38 and 103 unique to 100Gy, 140Gy and 200Gy irradiation respectively.

Further transcriptional analyses of the samples irradiated at 6 hours and 20 hours revealed a higher total number of differential gene transcripts at 20 hours when compared to 6 hours for 100Gy and 140Gy irradiated parasites (**Figure 5A**). For 100Gy irradiated parasites, 134 and 281 gene transcripts were upregulated whereas 100 and 141 were downregulated at 6 hours and 20 hours, respectively. At 140Gy irradiation, 184 and 259 were upregulated and 141 and 122 were downregulated at 6 hours and 20 hours, respectively. Interestingly, 200Gy irradiated parasites showed a reduced number of differential gene transcripts with 261 and 278 upregulated and 223 and 179 downregulated at 6 hours and 20 hours, respectively. In addition, the fold-changes observed at 20 hours were higher in upregulated transcripts when compared to those downregulated for 200Gy irradiated parasites (**Figure 5B**). The calculated individual FDR *p* values also tended to be more significant in the upregulated genes (**Figure 5B**).

When a comparison between 20 hours and 1 hour post-irradiation samples was made, 41.6% of all differential gene transcripts were shared between 100Gy, 140Gy, and 200Gy (ABC; 259 genes on Venn diagram) (**Figure 5C**). Other common differential gene transcripts were observed between 140Gy:100Gy (AB), 200Gy:100Gy (AC), and 200Gy:140Gy (BC) of 25, 36, and 59 respectively. The different doses also had unique differential gene transcripts with 102 for 100Gy, 38 for 140Gy, and 103 for 200Gy irradiation samples. An exhaustive comparison between differential gene transcripts across different doses and times post-irradiation is listed in **Table 1**. All doses showed a higher total difference at 20 hours apart from 200Gy which had its peak of differential expression at 6 hours. Interactions between different doses at different times post-irradiation were highest between 100Gy and 200Gy (**Table 1**). Gene lists for all doses and times post-irradiation with fold changes and FDR *p* values are attached as an excel file in the supplementary section (**Supplementary Table 2**). Further analyses that compared 100Gy- to 200Gy-irradiated parasites’ differential gene transcripts between 1 hour and 20 hours is provided in **Supplementary Table 4**. A delta fold change that describes the transcript concentrations of the affected transcripts at ±1.5-fold change was calculated to identify which genes rebound at 20 hours after irradiation between the two representative doses. A total of 75 genes had a + delta difference of 1.5 to 4.32, whereas 157 transcripts had a delta fold change of -1.5 to -3.27. A positive delta fold change designates a higher transcript concentration at 100Gy than 200Gy at 20 hours and inversely for a negative delta fold change (**Supplementary Table 4**).

**Table 1:** A comparison of gene transcripts across different doses and time.

Comparison	Down	Up	Total
200Gy (1h vs 6h)	223	261	484
200Gy (1h vs 20h)	179	278	457
100Gy (1h vs 20h)	141	281	422
140Gy (1h vs 20h)	122	259	381
1h vs 20h (all doses)	110	234	344
140Gy (1h vs 6h)	141	184	325
1h vs 6h (all doses)	109	138	247
100Gy (1h vs 6h)	100	134	234
200Gy vs 250Gy	125	71	196
0Gy vs 200Gy	28	134	162
140Gy vs 250Gy	84	57	141
100Gy vs 250Gy	85	46	131
140Gy (6h vs 20h)	3	128	131
0Gy vs140Gy	22	96	118
6h vs 20h	2	114	116
100Gy (6h vs 20h)	4	108	112
200Gy (6h vs 20h)	6	97	103
0Gy vs100Gy	10	85	95
100Gy vs 200Gy	0	47	47
6h (100Gy vs 200Gy)	0	46	46
140Gy vs 200Gy	0	33	33
0Gy vs 250Gy	6	22	28
1h (0 vs 250Gy)	6	22	28
1h (0 vs 200Gy)	8	15	23
20h (100Gy vs 200Gy)	0	18	18
1h (100Gy vs 250Gy)	2	11	13
6h (140Gy vs 200Gy)	0	8	8
1h (140Gy vs 250Gy	1	6	7
1h (0 vs140Gy)	2	4	6
1h (0 vs 100Gy)	2	3	5
1h (100Gy vs 200Gy)	2	2	4
100Gy vs 140Gy	0	4	4
20h (100Gy vs140Gy)	0	3	3
1h (140Gy vs 200Gy)	1	1	2
1h (100Gy vs 140Gy)	1	0	1
20h (140Gy vs 200Gy)	0	1	1
6h (100Gy vs 140Gy)	0	1	1
1h (200Gy vs 250Gy)	0	0	0
Interaction (1h and 6h vs 100Gy and 200Gy)	47	5	52
Interaction (1h and 20h vs 100Gy and 200Gy	22	6	28
Interaction (6h and 20h vs 100Gy and 200Gy)	8	10	18
Interaction (1h and 6h vs 140Gy and 200Gy)	14	1	15
Interaction (1h and 20h vs 100Gy and 140Gy)	7	4	11
Interaction (1h and 6h vs 100Gy and 140Gy)	9	1	10
Interaction (6h and 20h vs 140Gy and 200Gy)	2	4	6
Interaction (6 and 20 vs 100Gy and 140Gy)	2	2	4
Interaction (1 and 20 vs 140Gy and 200Gy)	3	0	3

**Figure 6:**
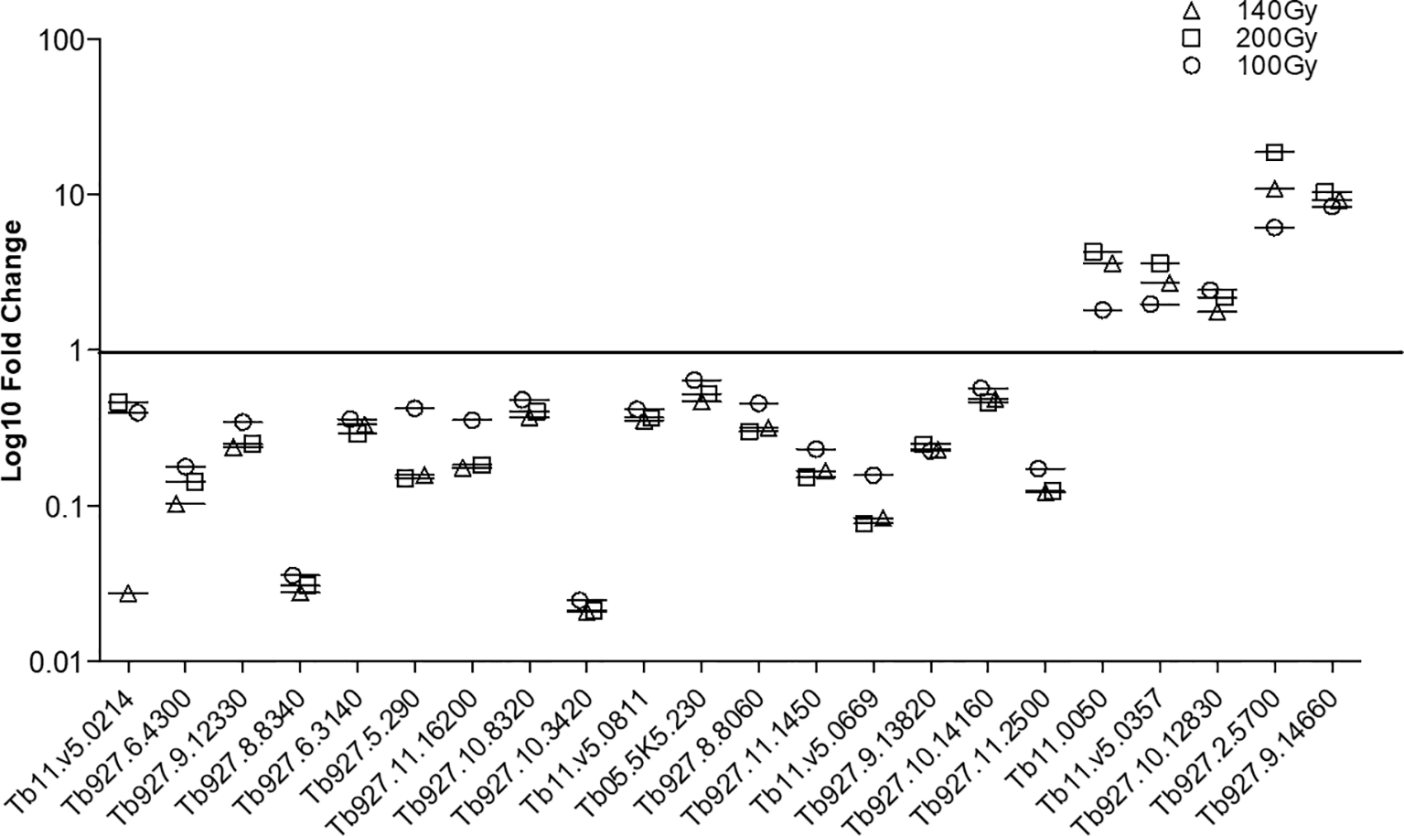
Confirmatory qPCR. Twenty-two genes that were either downregulated (17) or upregulated (5) in all three doses at 20h post-irradiation were amplified using the same RNA samples that had previously been processed using the TrypMS array.

In order to confirm the integrity of the TrypMS array, a confirmatory qPCR was performed on the same source of RNA used on the array (**Figure 6**). The targets chosen at 100Gy, 140Gy, and 200Gy irradiation had similar profiles with 17 downregulated and 5 upregulated genes in all three irradiation doses. The non-irradiated group (0Gy) was used as the baseline to calculate fold-change (**Figure 6**).

### The Effect of Irradiation on Gene Ontology

**Figure 7:**
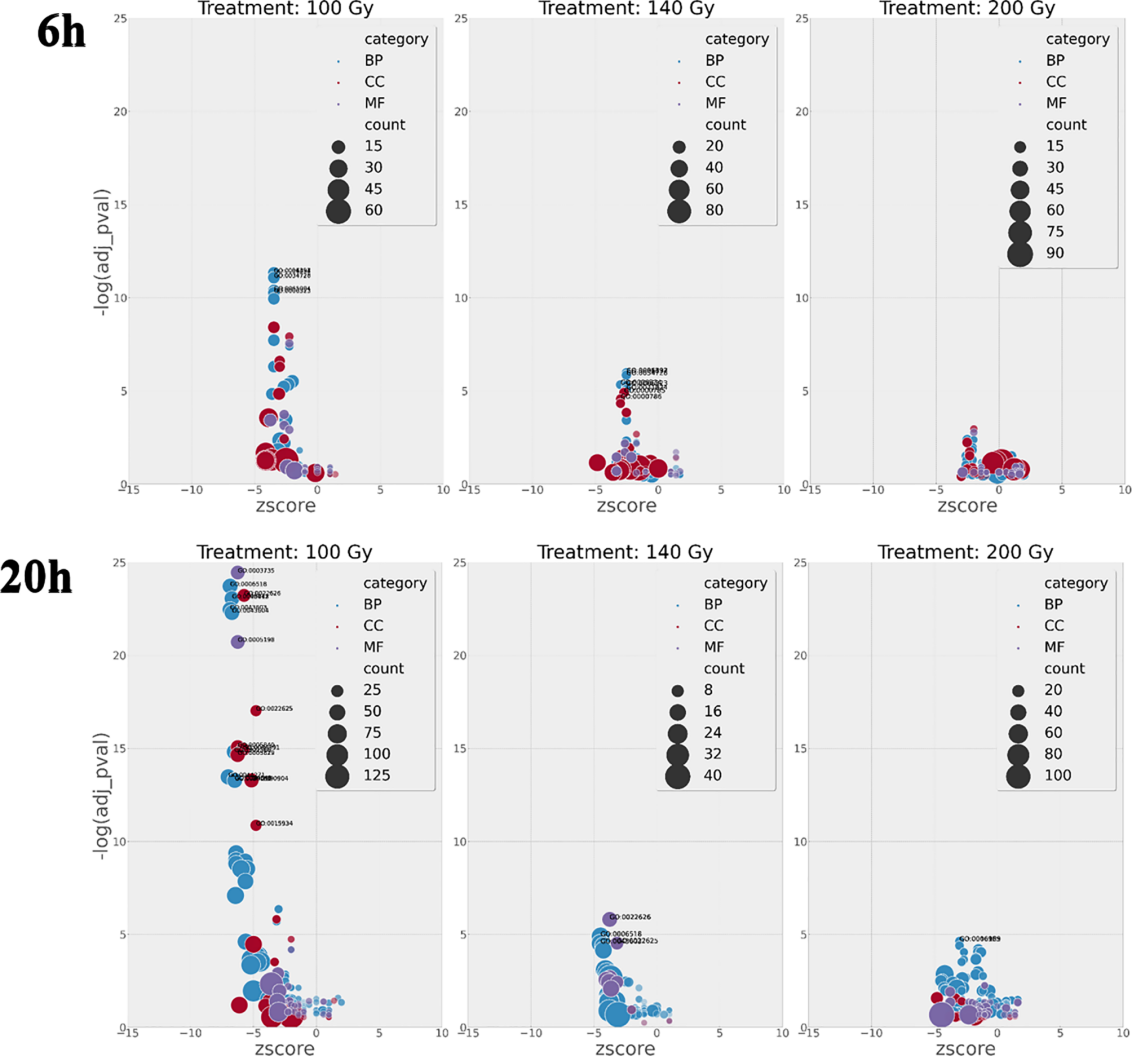
GO enrichment terms with calculated Z scores plotted against FDR *p* value at 6h and 20h hours post-irradiation. The number of terms increases exponentially at 100Gy irradiation after 6h and 20h when compared to 140Gy and 200Gy irradiation with negative log FDR *p* significance values 10 log higher at 100Gy 20h than 100Gy 6h. Adjusted *p* values are important as they designate if a process is more likely to occur.

Although the number of differential gene transcripts at all three doses was similar, ranging from 484 to 234 genes for 200Gy at 6h and 100Gy at 6h, respectively (**Table 1**), the effect on GO term enrichment was dramatically different both in the composition of GO terms affected and the significance in Z score (**Figure 7**). At 6h post-irradiation at 100Gy, GO terms associated with DNA packaging, chromatin assembly, and nucleosome assembly were significantly affected by irradiation. All top ten significant GO terms, except GO:0035328, had a decreasing Z score. A similar pattern was also observed at 140Gy and 200Gy, although with lower significance values at 5 logs and 8 logs less, respectively (**Table 2**). At 20 hours, GO terms associated with translation, ribosome structure, and peptide metabolic process feature prominently with *p* values 10 logs more significant when compared to GO terms affected at 6 hours (**Figure 7**). The GO terms with significantly adjusted *p* values associated with 140Gy at 20 hours are “cell periphery”, “plasma membrane”, “biological process involved in interaction with host”, “biological process involved in interspecies interaction between organisms” and “response to host and response to external biotic stimulus”. The GO terms with significant *p* values associated with 200Gy are “cell periphery”, “nucleoside diphosphate phosphorylation”, “nucleotide phosphorylation”, “nucleoside diphosphate metabolic process”, “purine nucleotide metabolic process” and “purine nucleoside diphosphate metabolic process”. A comprehensive description of all GO terms with associated gene IDs, logFC, adjusted *p* values and z scores at different irradiation doses and times post-irradiation (6 hours and 20 hours) is listed in **Supplementary Table 3**.

**Table 2:** Comparison of Go Term FDR *p* values across doses at 6h (top half) and 20h (bottom half) post-irradiation.

GO TERM	Description	100Gy Adj *p* value	140 Adj *p* value	200Gy Adj *p* value
GO:0006333	chromatin assembly or disassembly	1.5E-11	2.1E-06	1.6E-02
GO:0006334	nucleosome assembly	1.5E-11	8.4E-06	4.8E-02
GO:0031497	chromatin assembly	1.5E-11	2.1E-06	1.6E-02
GO:0034728	nucleosome organization	2.8E-11	2.8E-06	2.2E-02
GO:0065004	protein-DNA complex assembly	1.4E-10	5.9E-05	1.2E-01
GO:0006323	DNA packaging	1.8E-10	9.8E-06	5.2E-03
GO:0071824	protein-DNA complex subunit organization	3.7E-10	1.6E-05	6.7E-02
GO:0000785	chromatin	1.2E-08	2.5E-05	2.0E-02
GO:0035328	transcriptionally silent chromatin	2.0E-08	2.6E-03	1.4E-03
GO:0031490	chromatin DNA binding	4.7E-08	8.4E-03	2.1E-03
GO:0003735	structural constituent of ribosome	3.5E-25	N/A	1.2E-02
GO:0006518	peptide metabolic process	1.9E-24	1.3E-05	2.8E-03
GO:0022626	cytosolic ribosome	5.9E-24	1.6E-06	2.7E-02
GO:0006412	translation	8.8E-24	4.1E-05	1.0E-02
GO:0043043	peptide biosynthetic process	8.8E-24	4.1E-05	1.0E-02
GO:0043603	cellular amide metabolic process	3.4E-23	3.1E-05	3.2E-03
GO:0043604	amide biosynthetic process	5.0E-23	7.1E-05	7.5E-03
GO:0005198	structural molecule activity	1.9E-21	N/A	4.6E-02
GO:0022625	cytosolic large ribosomal subunit	9.4E-18	3.0E-05	4.0E-02
GO:0005840	ribosome	8.5E-16	2.7E-03	2.7E-01

N/A, Not Available.

**Figure 8:**
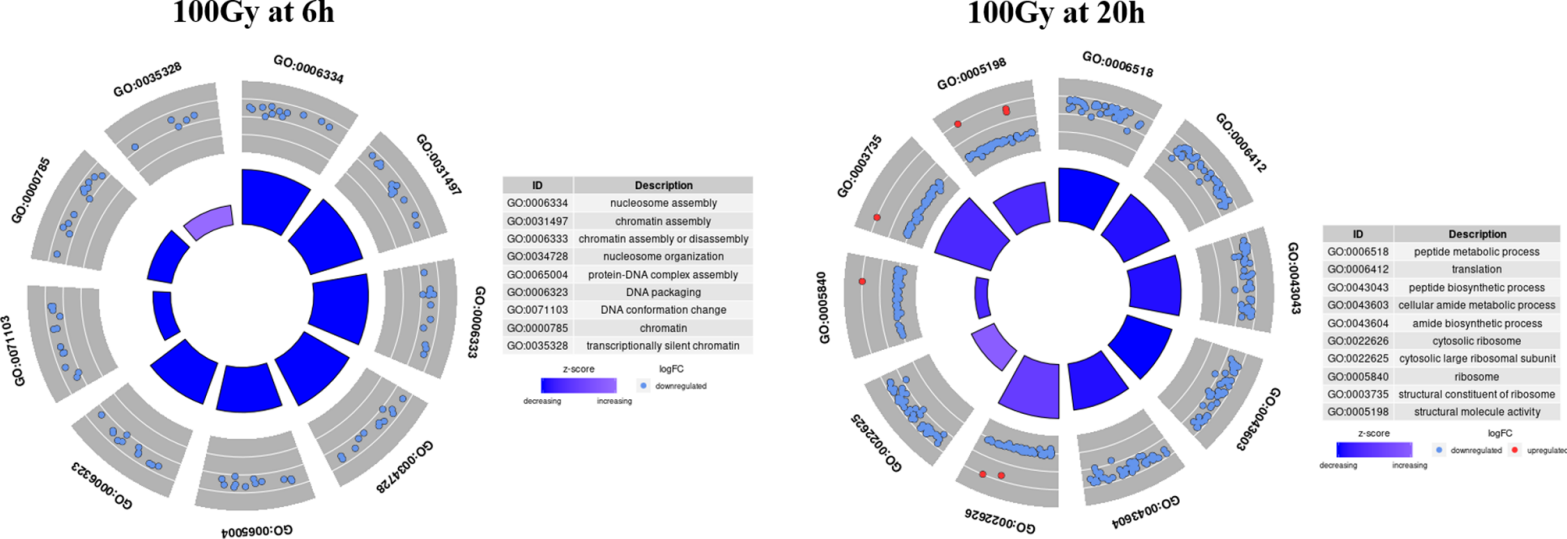
Top 10 most significant GO terms for 100Gy at 6h and 20h post-irradiation. GO circle plots for the top 10 most significant GO enrichment terms with fold-changes of individual genes taken in account and calculated as Z score at 20h post-irradiation. The height of the inner bar chat designates the significance of the GO term plotted. Most processes at both periods post-irradiation were down regulated.

GO circle plots plotted for the ten most significant enrichment terms for 100Gy at 6 hours shows the associated GO terms mostly have a decreasing Z score apart from G0:0035328; “transcriptionally silent chromatin” (**Figure 8**). However, at 20 hours post-irradiation, some of the most significant GO terms have an increasing Z score, e.g., “cytosolic ribosome” and “cytosolic large ribosomal subunit”; G0:0022626 and G0:0022625, respectively. A deeper look at the GO enrichment terms at 6 hours shows that the processes with the most significantly adjusted *p* values identified and most likely to occur, were all associated with the assembly and packaging of DNA and chromatin (**Table 2**). Similar processes were also found at 140Gy, although not in the top 10 (**Supplementary Table 3**). In addition, there were several logs of lower significance when compared to 100Gy where they featured at the topmost significant GO terms (**Figure 8**). The numbers were even lower when 200Gy was included in the comparison (**Table 2**). At 20 hours, the list of most significant GO processes changes to ribosomal structure, peptide metabolic, and peptide biosynthesis terms (**Table 2**, lower half). The top GO terms for 100Gy at 20 hours are also 10 logs more significant when compared to the top 10 terms at 6 hours at *p <*10^-20^ versus *p >*10^-11^,respectively. GO terms at 20h for 140Gy and 200Gy averaging with *p* > 10^-5^ and *p* > 10^-3^ are more than 20 logs less significant than the same terms at 20 hours post 100Gy irradiation (**Table 2**).

**Figure 9:**
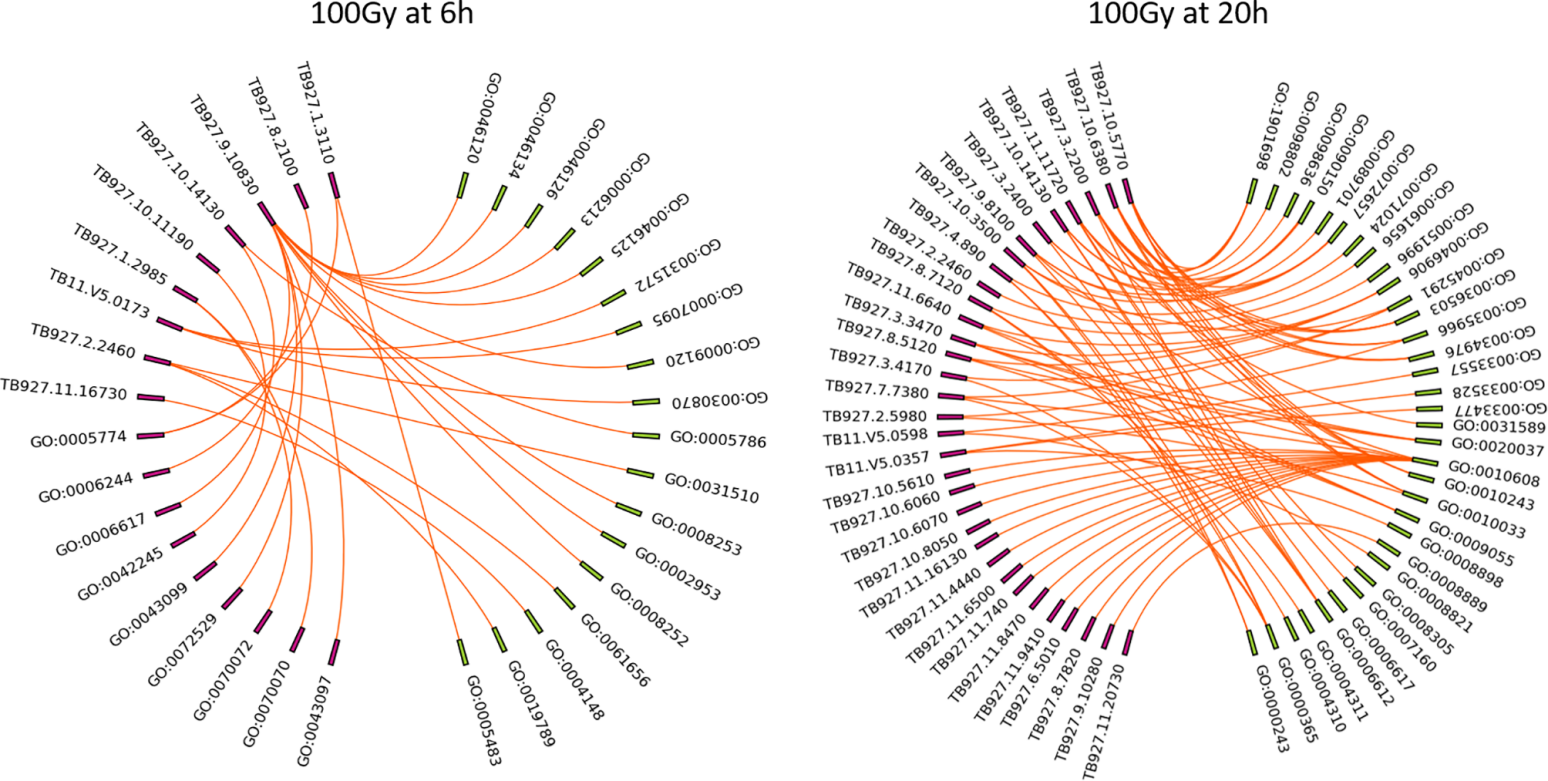
Chord plots for the positive Z score GO enrichment terms and associated genes at 6h and 20h post 100Gy irradiation. All the processes at 6h and 20h post-irradiation were plotted with corresponding genes to identify individual genes involved in multiple processes and given a chord plot count i.e., the higher the count a gene has, the more processes it will be involved with.

After identifying 100Gy as having a different response profile from the other doses, we identified the genes associated with the positive Z score GO terms (implying upregulation) at 6 hours and 20 hours after irradiation. A chord plot that links GO term to genes involved was constructed to select the most interactive genes and associated processes (**Figure 9**). None of the upregulated processes at 6 hours post 100Gy irradiation was associated with more than one gene, whereas a few genes were involved in multiple processes. For 20 hours, several genes and processes were associated with more than one entity (**Figure 9**). Genes were given a chord plot count ranked according to the fold-change difference between 6 hours and 20 hours (column 6, **Table 3**). Only two genes appeared at both times post-irradiation in the Z score positive GO terms (*Tb927.10.14130* and *Tb927.2.2460*; i and ii respectively in **Table 3**).

**Table 3:** Genes found in positive Z score GO terms for 100Gy at 6h and 20h post-irradiation.

Gene ID	Description	Chord plot count	6 vs 1 FC	20 vs 1 FC	6h vs 20h	Time FDR
Tb927.10.14130^i^	SRP19 protein, putative	2	1.69	1.77	0.08	2.07E-16
Tb927.11.16730	Dihydrolipoyl dehydrogenase	1	1.81	1.89	0.08	6.75E-18
Tb927.10.11190	tRNA-splicing ligase RtcB, putative	1	1.89	1.82	-0.07	4.44E-26
Tb11.v5.0173	Hypothetical protein, conserved	3	2.01	1.9	-0.11	1.32E-28
Tb927.1.3110	Soluble N-ethylmaleimide sensitive factor (NSF) attachment protein, putative	2	2.03	1.74	-0.29	2.09E-22
Tb927.9.10830	Metal dependent 5’-nucleotidase	13	2.07	1.75	-0.32	1.21E-29
Tb927.8.2100	Vacuolar ATP synthase 16 kDa proteolipid subunit, putative	1	2.43	2.07	-0.36	6.84E-18
Tb927.2.2460^ii^	Ubiquitin-conjugating enzyme E2, putative	3	2.84	2.38	-0.46	6.58E-18
Tb927.1.2985	ER protein Pkr1, putative	2	1.94	1.47	-0.47	4.87E-24
Tb927.8.7120	Squalene synthase, putative	3	-1.07	1.65	2.72	2.32E-08
Tb927.10.8050	TFIIF-stimulated CTD phosphatase, putative	1	-1.13	1.55	2.68	9.61E-08
Tb927.9.10280	Zinc finger CCCH domain-containing protein 48	1	-1.01	1.56	2.57	1.90E-06
Tb927.11.9410	Hypothetical protein	1	1.6	4.16	2.56	2.36E-17
Tb927.11.4440	Hypothetical protein	1	1.05	2.11	1.06	8.32E-17
Tb927.2.5980	Chaperone protein clpb1, putative	2	2.06	2.76	0.7	1.98E-17
Tb927.8.5120	Cytochrome c	3	-5.3	-4.62	0.68	1.51E-34
Tb11.V5.0357	Homocysteine S-methyltransferase, putative	3	1.09	1.76	0.67	7.40E-18
Tb927.11.8470	Zinc finger protein family member, putative	1	1.29	1.83	0.54	7.51E-05
Tb927.11.20730	Glycerophosphoryl diester phosphodiesterase, putative	1	1.36	1.87	0.51	7.19E-17
Tb11.V5.0598	GIY-YIG catalytic domain containing protein, putative	2	1.7	2.05	0.35	3.33E-26
Tb927.3.4170	CRK9-associated protein	2	1.8	2.13	0.33	4.30E-21
Tb927.11.6640	Cytochrome b5, putative	3	1.24	1.37	0.13	0.0302
Tb927.6.5010	Hypothetical protein, conserved	1	1.95	2.08	0.13	6.64E-28
Tb927.8.7820	‘Cold-shock’ DNA-binding domain containing protein, putative	1	2.18	2.31	0.13	1.92E-21
Tb927.10.14130^i^	SRP19 protein, putative	4	1.69	1.77	0.08	2.07E-16
Tb927.3.2200	TPR repeat, putative	8	1.58	1.66	0.08	7.27E-20
Tb927.4.890	Small nuclear ribonucleoprotein Sm D3	3	1.72	1.76	0.04	5.33E-15
Tb927.7.7380	U6 snRNA-associated Sm-like protein LSm3	3	1.96	2	0.04	2.51E-24
Tb927.10.5610	40S ribosomal protein S9, putative	1	-2.06	-2.04	0.02	1.84E-15
Tb927.10.3500	Splicing factor u2af 65 kDa subunit	4	2.09	2.1	0.01	7.08E-30
Tb927.11.11720	FG-GAP repeat protein, putative	5	1.87	1.86	-0.01	1.25E-23
Tb927.10.6380	Ring finger containing protein	6	1.83	1.81	-0.02	8.22E-17
Tb927.3.3470	Cytochrome b5, putative	4	1.91	1.84	-0.07	2.61E-19
Tb927.11.6500	40S ribosomal protein S21, putative	1	-1.74	-1.85	-0.11	1.35E-16
Tb927.3.2400	Palmitoyl acyltransferase 10, putative	3	2.34	2.22	-0.12	2.70E-25
Tb927.11.740	Eukaryotic translation initiation factor 5A	1	-1.59	-1.95	-0.36	9.17E-17
Tb927.10.6060	Universal minicircle sequence binding protein 2	1	-1.5	-1.89	-0.39	3.73E-10
Tb927.9.8100	Nascent polypeptide associated complex subunit, putative	4	-1.71	-2.1	-0.39	5.61E-24
Tb927.10.6070	Universal minicircle sequence binding protein 1	1	-2.32	-2.76	-0.44	7.22E-24
Tb927.10.5770	Valosin-containing protein	6	-1.59	-2.05	-0.46	1.34E-16
Tb927.2.2460^ii^	Ubiquitin-conjugating enzyme E2, putative	1	2.84	2.38	-0.46	6.58E-18
Tb927.11.16130	Nucleoside diphosphate kinase	1	-1.26	-2.1	-0.84	1.12E-23

i and ^ii^ found at both 6h and 20h.

## Discussion

Previous irradiation studies on trypanosome parasites began in the late 1960s with initial studies targeting *T. b. rhodesiense*, the sub-species responsible for acute trypanosomosis in humans (37). In the *T. b. rhodesiense* study, groups of mice were inoculated with 2 x 10^6^ irradiated parasites per animal using doses ranging from 100Gy to 1000Gy with some groups inoculated up to three times before challenge. After one round of inoculation, only mice inoculated with 100Gy irradiated parasites were infected and this dose was omitted in subsequent experiments. Interestingly, groups of mice inoculated twice with parasites irradiated at 200Gy and 400Gy had a 77% and 90% survival rate with no further mortalities after the third inoculation (37). Subsequent experiments using irradiated *T. b. rhodesiense* in cattle and rhesus monkeys were carried out using 600Gy irradiated parasites (38, 39, 44). In the current study, we used lower numbers of irradiated parasites per mouse and doubled the period between inoculations assuming that subsequent inoculations will not boost any undetectable infections. Similar to the study using *T. b. rhodesiense*, we also measured a dose-dependent delay in the prepatent period as the dose was increased to 200Gy (37). It may be argued that the number of 200Gy irradiated parasites used to inoculate mice was too low with only 6.3% of irradiated parasites surviving post-irradiation but it has previously been shown that as few as 100 - 200 *T. evansi* parasites are more than sufficient to establish an infection in female BALB/c mice (66). In addition, PBS control mice used in the study inoculated with 10^3^ parasites rapidly developed parasitemia. We therefore hypothesized that surviving 200Gy irradiated parasites *in vitro* lacked the parasite genes and processes described in 100Gy as necessary for establishing disease *in vivo*. All mice that survived inoculation, survived the subsequent homologous challenge as expected. This could be attributed to an efficient humoral response to the same circulating VSG previously encountered by the mice during inoculation (67).

Using 140Gy as a representative intermediate dose yielded interesting results, with two mice developing parasitemia after one inoculation and a third mouse after the second inoculation. The surviving five mice quickly developed parasitemia when compared to the remaining groups after the heterologous challenge. Previous infection and treatment studies with *T. evansi* that measured host responses after heterologous challenge displayed a prolonged prepatent period and partial protection in rabbits (68, 69). The contradictory results that we observed in mice inoculated with 140Gy irradiated parasites could possibly be explained by the immune- modulating effect of VSG in surviving mice that make them more susceptible to subsequent heterologous challenge (70). In the early stages of infection, carbohydrate moieties associated with soluble VSG released by circulating parasites induce TNF production by activated macrophages leading to trypanosome clearance. Dead parasites expose the VSG lipid moieties which cause overstimulation and subsequent TNF-mediated chronic inflammation in the host (70, 71). We speculate that initial inoculation with 140Gy irradiated parasites behaves similarly to 100Gy irradiated parasites for the three mice that succumbed before challenge, with the remaining five mice efficiently clearing parasites but left with high levels of circulating lipid-associated VSG. The subsequent heterologous challenge of these mice progresses similarly to a chronic infection with the new parasites causing more severe disease in the surviving 140Gy irradiated parasite-infected mice when compared to the PBS control group.

Cytokine profiles across the different irradiated parasite groups were irradiation dose-dependent with 0Gy, 100Gy, and 250Gy consistently producing higher levels of all cytokines apart from IL1a and TNFα when compared to 140Gy and 200Gy. It must be noted that the figures plotted are distributed across different time spans as mice inoculated with 0Gy- and 100Gy irradiated parasites survive for a much shorter period than those inoculated with 200Gy- and 250Gy irradiated parasites. Nevertheless, we are still able to estimate the general effect of irradiation. It was noted that mice inoculated with 140Gy irradiated parasites displayed a higher standard deviation when measuring Th1 type responses, mediated by IFNγ and TNFα which are essential when controlling the first peak of infection (72, 73). The deviation is exacerbated by the three mice that succumbed to parasitemia during the first peak and did not have the opportunity for Th2 type responses, mediated by IL10, IL13, IL4, and IL6 to develop. The mice inoculated using 200Gy irradiated parasites showed significantly lower values for the eight cytokines. This is possibly due to the parasites failing to establish disease since the host innate immunity was sufficient to clear the non-virulent parasites used for inoculation, especially in the case of *T. evansi* where type 1 responses are not required to control infection (74, 75). It has been well established in multiple previous studies that the products released by dead and dying Trypanosomes act as immune stimulators in the mammalian host (9, 76, 77). Because we inoculated mice with dead parasites at 250Gy, the products produced by this formulation were able to elicit high cytokine levels. In mice inoculated with 0Gy and 100Gy, mice were also exposed to these products as the initial immune Th1 response in inoculated mice killed off circulating parasites. This trend is however not as clear for 140Gy and 200Gy infected mice where the parasites do not for the most part establish an infection and do not release high amounts of parasite products in the host unlike 0-Gy, 100-Gy, and 250Gy irradiated parasites.

**Figure 10:**
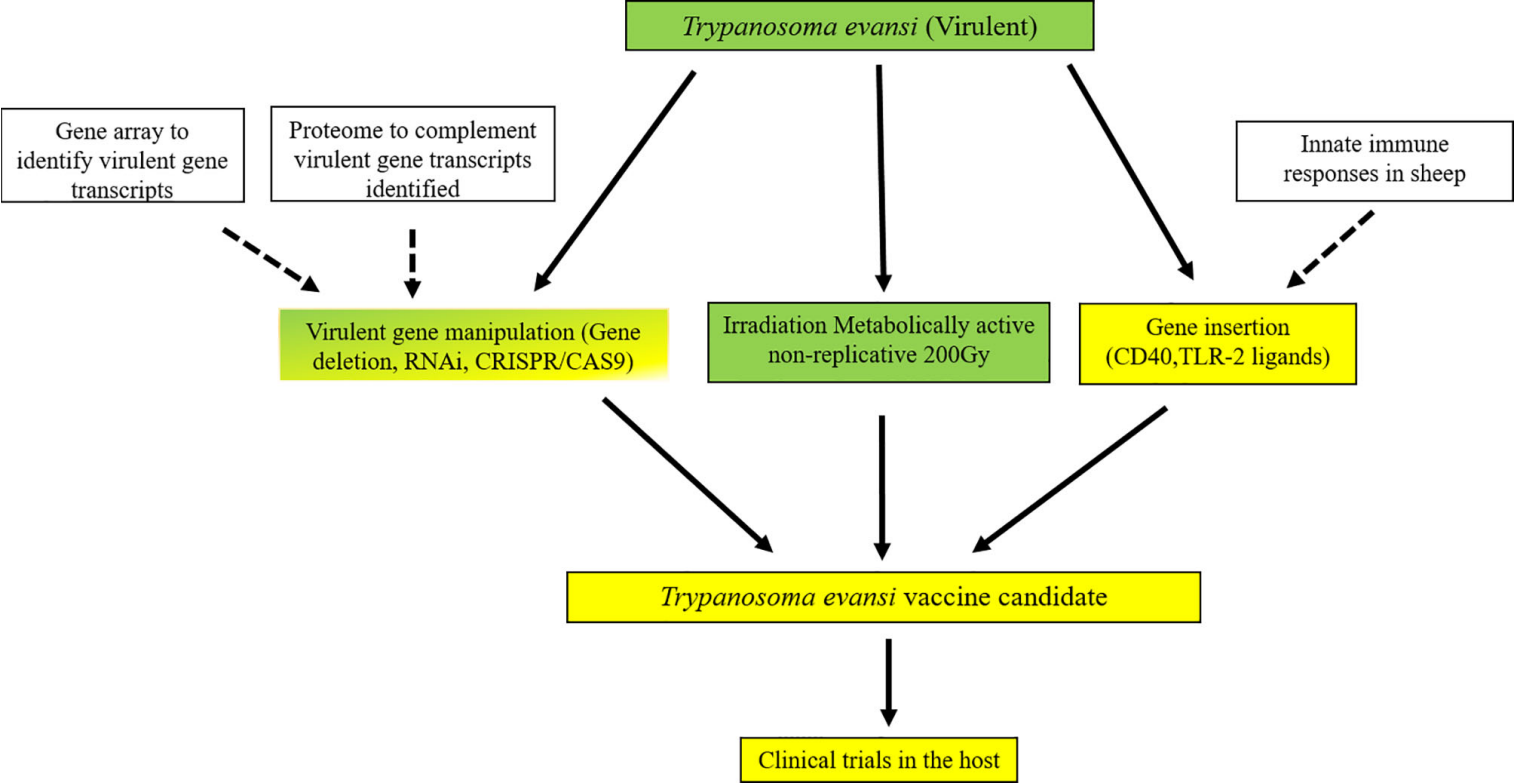
Potential pathway to an effective vaccine for *T. evansi* in livestock. An outline for developing a genetically manipulated and irradiated but metabolically active vaccine against Trypanosomosis in livestock species. Green signifies areas well studied, green/yellow areas are those that have been attempted, yellow are yet to be studied. White boxes are areas that are continuously under study that produce new information for vaccine development. All genes identified across doses were analysed in a matrix that considers the changes in the level of transcription at 100Gy compared to both 140Gy and 200Gy irradiation of *T. evansi* and functional analysis using gene ontology enrichment to identify pathways that are affected by low dose irradiation. Further analysis to look at immune functions of genes identified using gene deletion methods e.g., gene knock-out, RNAi or CRISPR/CAS9. Gene insertion of immune modulation ligands would further enhance immunogenicity of the whole parasite inoculum before testing in the mammalian host.

Following the *in vivo* characterization of 100Gy-, 140Gy, and 200Gy- irradiated parasites in mice, we further characterized the effect of irradiation on the parasites by measuring the transcript abundance of different parasite genes using the TrypMS array. We reasoned that since parasites irradiated at 100Gy successfully revived *in vitro* and can infect mice, they were probably able to quickly repair their damaged DNA and proceed to establish disease as critical genes required for both processes were still functional at 6 hours and 20 hours. Furthermore, it was established that these processes were also present in parasites subjected to a dose of 140Gy, albeit at a lower significance value. As the irradiation dose got closer to 200Gy, a much smaller number of parasites could sufficiently repair their DNA. None of the genes necessary to establish disease seemed to sufficiently increase in time to avoid host innate immune responses (21, 78). It could therefore be assumed that any processes with high significance at the 200Gy, 20 hour group are not crucial for establishing disease in mice. In contrast, those at 100Gy, 20 hours are necessary for the parasites after irradiation. Whereas most eukaryotes control the expression of genes *via* transcription initiation sites at the gene level, kinetoplastid gene expression regulation occurs post-transcriptionally with trypanosome RNA pol II transcribing poly-cistronic RNA transcripts that are processed by trans-splicing and polyadenylation to produce mature mRNA (79, 80). Due to the presence of untranslated RNA intermediates that exist during transcription, we refer to differentially regulated transcripts rather than genes because trypanosome mRNA concentrations do not correlate directly with the concentrations of protein translated (48). In addition, the stability, half-life, degradation, length and copy number of a transcript directly influence on the likelihood of translation (80). We therefore used a GO term approach that used RNA transcript levels to identify the most significant processes enriched at different irradiation doses with a corresponding Z score (64). As expected, the processes involved with DNA binding and chromatin assembly were the most important for 100Gy after 6 hours of irradiation, whereas those involved with ribosome structure and peptide biosynthesis processes at 20 hours. These processes remained significant for 140Gy irradiation at 6 hours and 20 hours but not at 200Gy. When looking at the genes involved in processes with a positive Z score at 6 hours and 20 hours at 100Gy irradiation, most genes identified at 6 hours are for nucleic acid assembly and repair. However, the transcripts identified at 20 hours have more varied functions and include genes such as TPR repeats and Squalene synthase which have been identified as a virulence factor in bacteria and a drug target in *T. cruzi*, respectively (81, 82). Further studies that elucidate the functions of these genes would help develop a genetically manipulated, non-infectious parasite that could potentially be used as a vaccine candidate in the mammalian host as described in **Figure 10**. If successful, an effective vaccine that includes genetically manipulated or irradiated parasites would require quality control measures to ensure safety due to the emergence of resistant parasites.

A comparison of gene transcripts from parasites irradiated at 100Gy and 200Gy at 20 hours with a delta fold change of ±1.5 identified eight genes that have been implicated in a study that described developmental gene regulation events that occur when non-infectious *T. brucei* procyclic insect forms in the tsetse gut and transform first into epimastigotes and subsequently to mammalian infectious metacyclic parasites in a processes known as metacyclogenesis (83). Metacyclogenesis in *T. brucei* can be replicated *in vitro via* the overexpression of RNA-binding protein 6 (RBP6) and has been modified further to skip the intermediate epimastigote stage by overexpressing either RBP6 with a single point mutation (Q109K), or RNA-binding protein 10 (RB10) in procyclic parasites (84–86). Of the eight transcripts identified in this study, one was a positive regulator of metacyclogenesis (Tb927.5.4000; hypothetical protein), and two were negative regulators (Tb927.10.9550 and Tb927.11.1310; hypothetical protein and NADH-cytochrome b5 reductase, putative). The remaining five were considered neutral and not affected by knocking out RBP6 in *T. brucei* procyclics (83). Interestingly, the one positive regulator of metacyclogenesis had a positive delta fold change of 1.67 signifying a higher number of copies in parasites irradiated at 100Gy (**Supplementary Table 4**).

Previous studies using irradiated *T. cruzi*, a trypanosome species that is highly resistant to irradiation, compared RNA transcripts to protein differences and found that both analyses complemented rather than confirmed each other (48, 49). A crucial role for the ubiquitin- proteasome system was also described during the repair process initiated after irradiation (87). In this study, ubiquitin-conjugating enzyme E2 is upregulated at both 6 hours and 20 hours post-irradiation at 100Gy. Surprisingly, although upregulated, DNA repair enzymes (RAD-51, BRCA2, MRE2, TOPO IIIα and RMI_1/2_) were not seen to be involved in the initial significant processes after irradiation (88–91). A more precise method of measuring RNA transcripts that are targeted for translation in trypanosome species is ribosome/polysome profiling (92). Ribosome profiling targets the parasite ‘translatome’ which are ribosome-bound mRNA or polysome when more than one ribosome is bound to the same mRNA moiety (93). The method begins with centrifuging a parasite lysate on a continuous sucrose gradient that separates polysomes from free mRNA ensuring that only ribosome-bound RNA is used for deep sequencing analysis. Fragmented unbound mRNA prepared from the same sample is also sequenced to act as a ribosome footprint library used to orientate sequenced polysomes (93). Although more accurate than total mRNA in terms of what is targeted for expression, it would still be prudent to analyze mRNA transcript levels to identify which target species exist in higher concentrations.

A *T. congolense* RNASeq study that compared parasite gene profiles during development in two distinct tsetse tissues, the cardia and proboscis, showed gene enrichment with GO terms associated with “quorum sensing”, “nucleotides”, “microtubules”, “cell membrane components” and “transport” in parasites colonizing the cardia (94). These terms were deemed essential for long non-dividing procyclic forms that colonize in the gut after a blood feed. Epimastigote and metacyclic forms that are found in the insect mouth parts on the other hand undergo cell division with enrichment terms associated with “nucleosomes”, “cytoplasm” and “membrane bound organelles” upregulated in preparation for infection. In addition, many hypothetical cell-surface proteins that have not been functionally characterized were also upregulated (94). When looking at the common terms that were upregulated in both insect parasite forms, “ribosome”, “cytosolic ribosomal unit”, “structural constituent of ribosome” and “translation” all featured prominently, irrespective of the tissue the parasite was isolated from (94). These terms also feature significantly in the 100Gy irradiated parasites after 20 hours, suggesting that they are important for constitutive processes in all forms of the parasites (**Table 2**). When looking at the most significant processes with a positive Z score, the terms “mRNA trans splicing, *via* spliceosome”, “commitment complex”, “protein targeting to membrane”, and “protein targeting to membrane” feature prominently (**Supplementary Table 2**, circ_100_20 hours), suggesting that these processes are unique to post-irradiation, damage repaired parasites possibly upgrading these processes for establishing disease utilizing specific genes identified and displayed in **Table 3**.

In summary, we irradiated *T. evansi* parasites to identify doses sufficient to produce viable parasites *in vitro* but are defective in causing infection in BALB/c mice. with irradiation doses of 200Gy, 140Gy, and 100Gy producing representative non-infectious, intermediate, and infectious parasites, respectively. Interleukin analyses of inoculated mice revealed that 140- and 200Gy irradiated parasites did not mediate a Th1 response in the host as is expected in regular infection studies. To further characterize the irradiated parasites, we developed a multispecies micro-array, TrypMS, to measure mRNA abundance across the three representative doses at 6 hours and 20 hours post-irradiation. The information gathered from TrypMS was then used to calculate Z scores using a gene ontology approach that measured the enrichment of processes rather than accessing individual genes. Transcript fold changes were also compared between 100- and 200Gy parasites at 20 hours post-irradiation to predict genes that may be involved in establishing an infection after parasite irradiation repair. A list of genes from GO processes upregulated at 20 hours by 100Gy irradiated parasites was also generated for further study and the pathway to a functional irradiated vaccine postulated.

## Data Availability Statement

Microarray data produced from this experiment is available in the ArrayExpress database (http://www.ebi.ac.uk/arrayexpress) under accession number E-MTAB-11705. The TrypMS521152F custom array from Affymetrix has the accession number A-MTAB-695 (95).

## Ethics Statement

The animal study was reviewed and approved by institutional ethics committee of the University of Veterinary Medicine, Vienna, and the national authority according to § 26 of the Austrian Law for Animal Experiments, Tierversuchsgesetz 2012-TVG 2012 under the No. GZ 68.205/0069-WF/II/3b/2014.

## Author Contributions

RK, EW, and AD conceptualized the work; RK, EW, TS, and HU carried out the experiments; RK, SD, VW, and CL analyzed the data. All authors contributed to the article and approved the submitted version.

## Conflict of Interest

The authors declare that the research was conducted in the absence of any commercial or financial relationships that could be construed as a potential conflict of interest.

## Publisher’s Note

All claims expressed in this article are solely those of the authors and do not necessarily represent those of their affiliated organizations, or those of the publisher, the editors and the reviewers. Any product that may be evaluated in this article, or claim that may be made by its manufacturer, is not guaranteed or endorsed by the publisher.

## Supplementary Material

Supplementary Figure 1 Gating strategy for Trypanosome CFSE replication assay.

Supplementary Figure 2 Calculated D10 for *T. evansi* RoTat 1.2 parasites.

Supplementary Figure 3
*In vitro* parasite counts post-irradiation. **(A)** Percentage number of wells with viable parasites in a 24 well plate at different doses. **(B)** Log parasite numbers where more than 25% or more of wells in 24 well plates contained motile and viable parasites at different doses.

Supplementary Table 1 Primer list for 22 genes used to confirm expression.

Supplementary Table 2 Gene transcripts at different times and doses post-irradiation.

Supplementary Table 3 Combined GOcirc data.

Supplementary Table 4 Differential gene transcripts between 1h and 20h for 100Gy- to 200Gy-irradiated parasites.
