# Supplementary figures and images for: Low Dose Gamma Irradiation of Trypanosoma evansi Parasites Identifies Molecular Changes That Occur to Repair Radiation Damage and Gene Transcripts That May Be Involved in Establishing Disease in Mice Post-Irradiation
Source: Front Immunol. 2022 May 13;13:852091. doi: 10.3389/fimmu.2022.852091 (PMC9136415; doi:10.3389/fimmu.2022.852091)

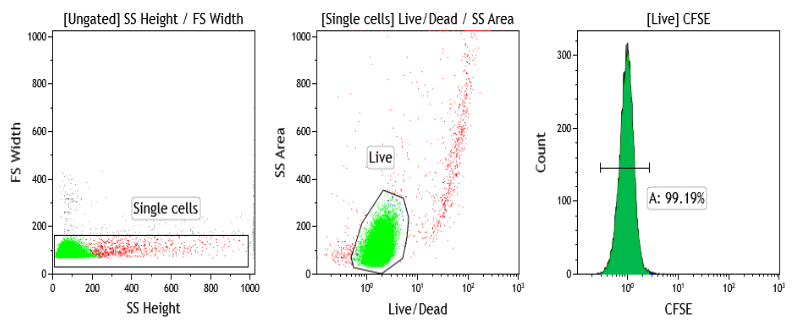

Supplement: Supplementary Figure 1 — Gating strategy for Trypanosome CFSE replication assay. [file Image_1.tif]

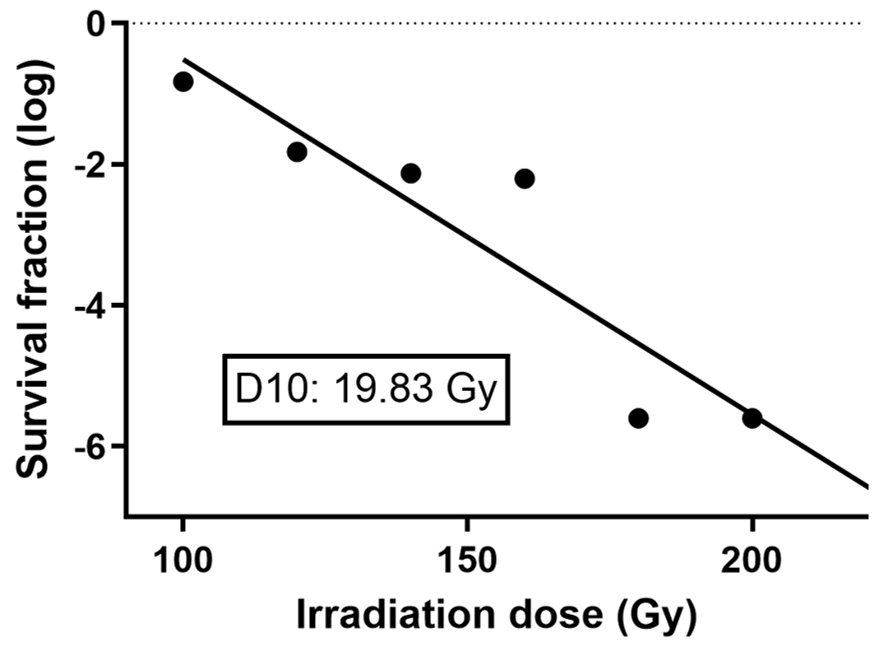

Supplement: Supplementary Figure 2 — Calculated D10 for T. evansi RoTat 1.2 parasites. [file Image_2.tif]

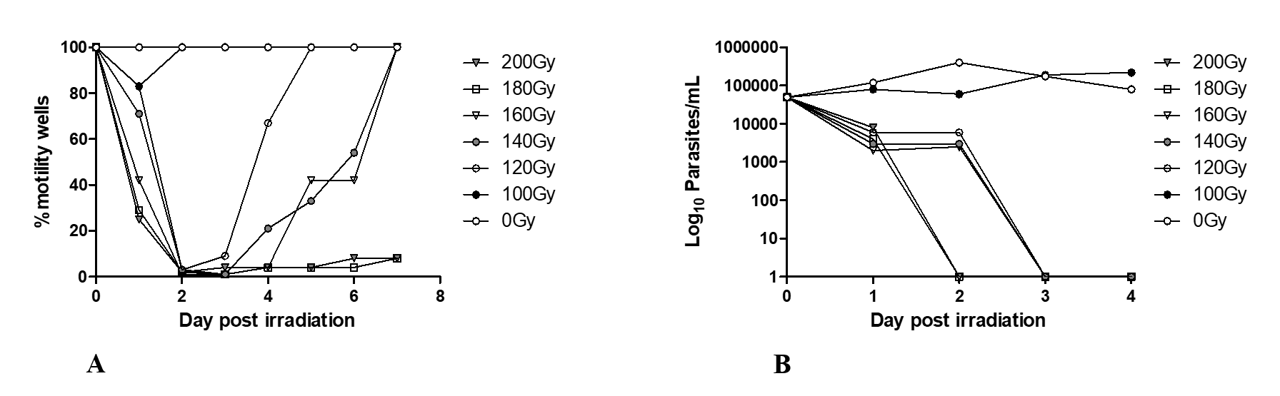

Supplement: Supplementary Figure 3 — In vitro parasite counts post-irradiation. (A) Percentage number of wells with viable parasites in a 24 well plate at different doses. (B) Log parasite numbers where more than 25% or more of wells in 24 well plates contained motile and viable parasites at different doses. [file Image_3.tif]
